# Role of Questionnaires in the Assessment of Severity and the Outcomes of Minimally Invasive Surgery for Snoring and Obstructive Sleep Apnea

**DOI:** 10.3390/jcm14155268

**Published:** 2025-07-25

**Authors:** Natalia Olszewska, Ewa Olszewska, Cuneyt M. Alper

**Affiliations:** 1 Student Research Group, Department of Otolaryngology, Faculty of Medicine, Medical University of Bialystok, 15-089 Bialystok, Poland; nataliaolszewska861@gmail.com; 2 Department of Otolaryngology, Sleep Surgery Center, Faculty of Medicine, Medical University of Bialystok, 15-276 Bialystok, Poland; 3 Department of Otolaryngology, University of Pittsburgh School of Medicine, Pittsburgh, PA 15213, USA

**Keywords:** obstructive sleep apnea, sleep questionnaires, sleep study, sleep surgery

## Abstract

**Background/Objectives**: Sleep questionnaires are used as screening tools to estimate the presence and severity of snoring and obstructive sleep apnea (OSA). The aim was to prospectively assess the diagnostic and prognostic accuracy of sleep questionnaires (Epworth Sleepiness Scale (ESS), Visual Analog Scale for snoring loudness (VAS), Short Form Health Survey 36 (SF-36), STOP-Bang, and Pittsburgh Quality of Sleep (PSQI)) in subjects who underwent minimally invasive surgery for snoring and OSA. **Methods:** A total of 49 participants with primary snoring and/or OSA underwent minimally invasive surgery. Pre- and post-operative sleep study parameters and sleep questionnaire results were analyzed to assess the correlation between the subjective and objective parameters before and after surgery and changes with the surgery. **Results:** Pre-operative sleep study parameters demonstrated: an apnea–hypopnea index (AHI) of 16.71 ± 9.31, oxygen desaturation index (ODI) of 14.43 ± 9.31, and mean percentage of snoring time (ST) of 17.26 ± 14.5%, ESS of 9.04 ± 5.76, VAS of 8.18 ± 1.93, SF-36 of 42.12 ± 22.86, STOP-Bang of 3.65 ± 1.13, and PSQI of 6.61 ± 3.23. Post-operative sleep study parameters demonstrated an AHI of 10.39 ± 7.86, ODI of 10.17 ± 7.78, and ST of 12.55 ± 13.36%, ESS of 6.61 ± 4.55, VAS of 4.13 ± 2.87, SF-36 of 42.45 ± 24.70, STOP-Bang of 2.49 ± 1.42, and PSQI of 4.98 ± 2.13. Changes with surgery for sleep parameters demonstrated a decrease in AHI: 37.83%, ODI: 29.52%, ST: 27.3%, ESS: 26.86%, VAS: 49.50%, PSQI: 24.69%, and STOP-Bang: 31.84%. The score of SF-36 was not significant. **Conclusions:** Sleep questionnaires are an essential component of the workup for patients with snoring and OSA. There are differences in their ability to identify the presence and quantify the severity of snoring and OSA when compared to objective sleep parameters. Their sensitivity in assessing changes with treatment also varies.

## 1. Introduction

Snoring and obstructive sleep apnea are highly prevalent manifestations of sleep disturbance. Snoring can be defined as a rough, rattling noise made by turbulent airflow mainly during inspiration. It occurs during sleep and is caused by the vibration of soft tissues in the upper airways. Obstructive sleep apnea syndrome (OSA) is a sleep disorder that involves cessation or a significant decrease in airflow in the presence of breathing effort. Snoring is often only considered to be an annoyance to bed partners and other household members. However, snoring has significant physical and social consequences. The prevalence of OSA is 10% of men and 3% of women between the ages of 30–49, increasing to 17% and 9% between the ages of 50–70, respectively [1]. Meanwhile, the prevalence of snoring is estimated as 35–45% in men and 15–28% in women, and increases with age [2,3]. It is estimated that globally, 1 billion people are affected by sleep apnea [4]. Even though snoring accompanies most OSA cases, it is much more prevalent. While the presence and severity of snoring may be known to the patient’s bed partners and consequently to the individual, most patients do not seek an assessment of treatment. Therefore, OSA is often underdiagnosed, and its negative effects are undervalued. It has been estimated that up to 80% of individuals with moderate-to-severe OSA may remain undiagnosed [5]. Undiagnosed and untreated OSA has a major impact on an individual’s health, with significant potential morbidity and mortality. Moreover, it causes major social and economic costs to society [6,7]. Accurate diagnosis of OSA requires a sleep study. However, considering limited resources, only those individuals who are suspected to have sleep-disordered breathing may end up participating in a sleep study. Due to the huge discordance between the prevalence and the ability to access sleep studies, there is a serious need for accurate tools to screen and identify individuals at risk for OSA.

Questionnaires are developed to estimate the presence and severity of snoring and OSA. They are used as screening tools to assess the personal and social impact of previously mentioned conditions, as well as the quality of an individual’s life. Moreover, questionnaires may be used in diagnostics as auxiliary tools, as well as in monitoring the progress and response to any treatment. The management of snoring and OSA, particularly when considering surgical intervention, benefits greatly from a multidisciplinary team (MDT) approach. Collaboration among sleep physicians, otolaryngologists, pulmonologists, cardiologists, maxillofacial surgeons, and sleep technologists ensures comprehensive assessment, appropriate decision-making in the diagnostic process, and individualized treatment planning. Questionnaires serve as valuable tools within this framework, helping to standardize symptom evaluation and monitor outcomes across disciplines. Incorporating the expertise of an MDT enhances the interpretation of subjective and objective data, ultimately contributing to improved patient care and surgical outcomes.

However, the accuracy of questionnaires as screening, diagnostic, and prognostic tools is not well known. It is also unclear which questionnaires are more sensitive and/or specific for snoring and/or OSA.

Therefore, the study aimed to prospectively assess the diagnostic and prognostic accuracy of the subjective sleep parameters (obtained from sleep questionnaires) in subjects who underwent minimally invasive surgery for snoring and obstructive sleep apnea syndrome (OSA) by comparing them to objective sleep parameters (obtained from sleep studies). We believe that this study is the first one evaluating all five questionnaires together in the context of minimally invasive surgery for snoring and OSA.

## 2. Materials and Methods

Patients seen in the outpatient clinic with concerns about snoring and/or OSA underwent evaluation, including obtaining a detailed general and snoring/OSA-related history. Patients had a general and ENT examination, a flexible nasolaryngoscopy, and a type III sleep study (SOMNOtouch^TM^ Resp, SOMNOmedics GmbH, Randersacker, Germany). For those who were diagnosed with OSA, a titration study for positive airway pressure (PAP) treatment was offered. Those patients who did not receive, accept, or fail the PAP treatment were considered to undergo palatoplasty and tongue base volume reduction under local anesthesia. If indicated, inferior turbinate reduction using radiofrequency (RF) ablation was also performed. Patients scheduled for minimally invasive procedures under local anesthesia were screened, and those who met the inclusion and exclusion criteria were invited to enroll in this study.

Eligibility criteria included age between 18 and 75, history of snoring and mild to severe OSA in sleep study, body mass index (BMI) less than 35, no concomitant diseases, no severe gag reflex, and having not met any exclusion criteria. Exclusion criteria included central sleep apnea syndrome, severe obesity (BMI ≥ 35), history of treatment for sleep apnea within 3 months before enrolment, history of respiratory infection within previous 4 weeks, history of rheumatic diseases, coagulation disorders, acute or chronic kidney failure (renal failure defined as serum creatinine > 2.0 mg/dL), a history of injury or surgery in 3 months before enrolment, presence of other respiratory diseases (such as asthma or chronic obstructive pulmonary disease) based on clinical history, severe cardiovascular disease by history, systemic inflammatory diseases, diabetes, comorbidities that may affect systemic inflammation (such as collagen vascular disease or cancer), chronic rhinosinusitis, receiving medical treatment that include hormones, immune suppressors, cytotoxins, or free radical scavengers.

The parameters included in the analysis of the type III sleep study results included apnea and hypopnea index (AHI) as the number of apneas and hypopneas per hour of sleep, mean oxygen saturation (MOS), lowest oxygen saturation (LOS) as percent value of lowest oxygen saturation, oxygen desaturation index (ODI) as the average number of desaturation episodes per hour of sleep, snoring time (ST) as percent time of sleep with snoring, and percent of sleep time with the oxygen saturation below 90% (TID).

The surgery was performed under local anesthesia in an outpatient sleep center. Subjects were asked not to eat or drink anything for 2 h prior to the surgery. On the day of surgery, subjects had a review of their history for verification, were examined again, received specifics of the procedure, risks, and anticipated outcomes, and recommended post-operative care and follow-up were reviewed. Upon confirming and obtaining surgical consent, an IV line was established, and first, topical anesthesia was administered using 10% lidocaine spray (6 applications: 4 on the mucosa of the soft palate and uvula and 2 on the central part of the base of tongue). Second local anesthesia was administered using 3% Mepivacaine that was injected in the amount of 0.5 mL in the area of 1 cm above the base of the uvula, 0.5 mL on both sides 1 cm from the midline injection and in the amount of 1 mL to the tongue base (in front of the midline circumvallate papillae). If planned, 0.5 mL was also applied submucosally to the inferior turbinates. The radiofrequency (RF) device (Micromed RF130, Wurmlingen, Germany) with the setting of 4 MHz delivered about 70° heat to the target tissues. The RF probe was first applied to the soft palate in 3 locations: 1 cm above the base of the uvula, 1 cm to the right, and to the left of the first location for 5–12 s each. Then, the RF was inserted into 6 locations at the base of tongue: 3 anteriorly and 3 posteriorly from the circumvallate papillae (in the midline and 1–1.5 cm laterally from the midline). The 45° or 30° endoscope was helpful for better visualization of the base of tongue. After observation for approximately 1 h, subjects were sent home with medical management instructions. Subjects were re-evaluated a minimum of 2 months after the surgery. The sleep study was repeated, and all subjects completed all 5 previously mentioned questionnaires as part of the follow-up workup.

### Statistical Analysis

Descriptive statistics reporting in the manuscript included sleep parameters pre-operatively and post-operatively, for subjective sleep parameters (sleep questionnaires) including 5 different questionnaire scores, and for the objective sleep parameters (sleep study) including 8 sleep study parameters. Each of these parameters is presented as the number of subjects, average, standard deviation, minimum, quartile 1, median, quartile 3, and the maximum value (Table 1 and Table 2).

Statistical comparison of pre- and post-operative values of the subjective sleep parameters and objective sleep parameters (sleep study) was conducted using the Wilcox pairwise order test, with the *p* value set as <0.5. This non-parametric test was suitable for the analysis of the paired data of pre- and post-operative median values of the same subjects (Table 3 and Table 4).

Statistical correlation among the subjective and objective sleep parameters was conducted independently utilizing non-parametric Spearman correlation analysis with *p* < 0.5. This statistical method was selected as the current data is not normally distributed. This analysis focused on the correlation between parameters, pre-operatively, post-operatively, and lastly between the preoperative values and the magnitude of difference between pre- and post-operative values for each parameter (Table 5, Table 6 and Table 7).

Gender specific differences, differences as per BMI categories, and AHI categories were also statistically analyzed. Comparison of each of these categorical variables was conducted separately for pre-operative and post-operative data utilizing the Wilcoxon Signed Rank Test. Additionally, for each category, comparison of pre-operative and post-operative values was conducted utilizing the Mann–Whitney/Kruskal–Wallis test. *p*-value was set as <0.05 for both analyses (Table 8, Table 9 and Table 10). Correlations among the subjective sleep parameters only were analyzed for the pre-operative and post-operative values independently, again utilizing non-parametric Spearman correlation analysis, with the *p*-value < 0.05 (Table 11 and Table 12). We used the strength of the correlation based on the absolute value of the positive/negative correlation coefficient r as: very weak when the value was 0.00 to ±0.19, weak: ±0.20 to ±0.39, moderate: ±0.40 to ±0.59, strong: ±0.60 to ±0.79, and very strong: ±0.80 to ±1.00. We applied the Bonferroni correction in relevant analyses.

## 3. Results

A total of 49 subjects (39 male, 10 female) with a mean age of 40.6 ± 11.5 (range: 24–71) years old were enrolled. Average weight and height were, respectively, 85.7+/−14.4 kg (range: 52–116 kg) and 176.8+/−8.6 cm (range: 161–192 cm. Average body mass index (BMI) was 27.3+/−3.3 (range 20.1–32.7). The BMI of 14 subjects was less than 25, 26 patients’ BMIs fluctuated between 25 and 29.9, and 9 patients had a BMI that was greater than 30.

All subjects received standardized questionnaires (translated into Polish): Epworth Sleepiness Scale (ESS) [8], the visual analog scale for snoring loudness (VAS) [9], Short Form Health Survey 36 (SF-36) [10], STOP-Bang [11] (stands for questions regarding snoring, tiredness, observed apnea, high blood pressure, body mass index, age, neck circumference, and gender), and Pittsburgh Sleep Quality Index (PSQI) [12].

A total of 35 subjects (71.4%) had no co-morbidities or risk factors. A total of 14 remaining patients (28.6%), 7 (14.3%) suffered from hypertension, 2 (4.1%) had diabetes, 1 (2%) hypothyroidism, 3 (6.1%) depression, and 8 (16.3%) were smokers. On exam, 4 patients (8.2%) had no tonsils; 36 patients (73.5%), 4 (8.2%), and 5 (10.2%) had grade I, I/II, and II tonsil size according to the Friedman palatal tonsils scale, respectively [13]. The base of tongue size distribution was 1 (2%), 1 (2%), 2 (4.1%), 10 (20.4%), 3 (6.1%), 6 (12.2%), and 25 (51%) for grade I, II, IIA, IIB, IIA/IIB, IIB/III, and III, respectively [13]. Nasal septum was deviated in 37 subjects (75.5%), inferior turbinates were enlarged in 37 subjects (75.5%). Inferior turbinate enlargement was present in 37 subjects (75.5%).

Table 1 shows pre-operative results for sleep questionnaires (ESS, VAS, SF36, STOP-Bang, PSQI) and sleep study parameters (AHI, MOS, LOS, ODI, ST, and TID). The table also displays the number of subjects, the average, standard deviation, minimum and maximum numbers, the median, and the interquartile range.

There were no intraoperative complications. Post-operatively, palatal edoema was observed in six subjects, transient palatal hematoma in one patient that resolved without a problem, and mild swallowing problems were seen in three patients. There were no other complications.

Postoperative sleep questionnaire results and sleep study results for all similar parameters are shown in Table 2.

Comparison of the subjective sleep parameters, i.e., the sleep questionnaire results before and after the surgical treatment of snoring and OSA, is demonstrated in Table 3. The results of the questionnaires: ESS, VAS, PSQI, and STOP-Bang improved significantly after the surgery. The greatest change was observed in VAS and STOP-Bang results. The SF-36 questionnaire did not show a significant change.

Comparison of the objective sleep parameters, i.e., sleep study results before and after the surgical treatment of snoring and OSA, is shown in Table 4. AHI and ODI improved significantly after the surgery, though AHI had relatively higher statistical significance. The sleep study parameters MOS, LOS, ST, and TID did not change significantly.

Correlations between the results of subjective and objective sleep parameters before the surgery are shown in Table 5. Only a few correlations between these parameters were significant. STOP-Bang showed a weak correlation with AHI (r = 0.29) and ODI (r = 0.34). Weak negative correlations were observed between SF-36 and ST (r = −0.38) and between PSQI and ST (r = −0.35). Questionnaires ESS and VAS demonstrated very weak correlations with sleep parameters.

Correlations between the results of subjective and objective sleep parameters after the surgery are shown in Table 6. A greater number of significant correlations between these parameters was observed. STOP-Bang showed a weak positive correlation with AHI (r = 0.30) and ODI (r = 0.31) and a weak negative correlation with LOS (r = −0.39). VAS exhibited a weak negative correlation with LOS and a weak positive correlation with TID (r = 0.29). The remaining questionnaires (ESS, SF36, and PSQI) demonstrated very weak correlations with the sleep parameters.

Statistical analysis of the correlations between the pre-operative subjective sleep parameters and the magnitude of change in the objective sleep parameters with the surgical treatment of snoring and OSA is shown in Table 7. There was only a correlation observed between the VAS questionnaire and two sleep study parameters: a weak negative with LOS (r = −0.31) and a weak positive with TID (r = 0.30).

Gender-specific differences in the sleep parameters before the surgery demonstrated significant differences in values of VAS, AHI, and ODI (Table 8). Post-operatively, differences between females (*n*: 10) and males (*n*: 39) were significant only for ODI. Comparison of pre- and post-operative values of sleep parameters was significant in both females and males for ESS and VAS. Pre- and post-operative values of STOP-Bang, PSQI, AHI, and ODI were significantly different only in males.

Differences in sleep parameters findings as per BMI categories were investigated for the categories defined as <25 (*n*: 14), 25–29.9 (*n*: 26), and 30 and over (*n*: 9). Pre-operatively there were statistically significant differences between the categories only for MOS (Table 9). Post-operatively, differences between BMI categories were not seen in any parameter. Differences between the pre- and post-operative values of sleep parameters were significant in all BMI categories, only for VAS. Statistically significant differences were observed in two categories of BMI, equal to or more than 25, for ESS, STOP-Bang, and PSQI. Significant differences were seen in two BMI categories under 30 (<25 and 25–29.9) only for AHI. The changes with surgery were significant in ODI only for the middle BMI category of 25–29.

Results were also analyzed separately for different AHI categories of <5 (*n*: 5), 5–14.9 (*n*: 20), 15–29.9 (*n*: 20), and 30 and over (*n*:4). Pre-operatively there were no statistical differences between the categories in any of the questionnaires (Table 10). Pre-operatively, there were significant differences between the AHI categories for all sleep study parameters except ST. Similarly, there were no differences between AHI categories post-operatively. Sleep study parameters were statistically significant between AHI categories for AHI, LOS, and ODI. Differences between the pre- and post-operative values of sleep parameters were significant in the two middle AHI categories (5–14.9 and 15–29.9) for VAS, STOP-Bang, PSQI, and AHI. For the first category of AHI ( < 5), changes with surgery were significant for SF-36 and ST. In the AHI category of 5–14.9, statistically significant changes with surgery were present for ESS. For the AHI category of 15–29.9 significant change with surgery was seen in ODI.

Pre-operatively, both STOP-Bang and PSQI showed a weak positive correlations with SF-36 (Table 11). Post-operatively, except for the correlations between PSQI and ESS (weak, r = 0.20), between PSQI and VAS and SF-36 and VAS, all questionnaires demonstrated higher strength of positive correlations. A strong positive correlation was observed between STOP-Bang and VAS (r = 0.69) (Table 12).

## 4. Discussion

The individual would not be the source of information in a questionnaire to qualify or quantify snoring and/or OSA that they are experiencing during sleep. They would simply not be aware of what was happening during their state of sleep. On the other hand, the individual may provide information related to their sleep based on either the feedback they have received from others or by addressing a set of surrogate markers that they may be aware of.

In evaluating the outcomes of minimally invasive surgery for snoring and OSA, both objective sleep parameters and subjective questionnaire data provide valuable, yet distinct, insights. While objective sleep parameters, such as AHI, are essential for diagnosing OSA and differentiating the severity of OSA, they do not always reflect patient-perceived improvements. In our opinion, validated questionnaires should play a higher role in assessing treatment outcomes, as they capture symptoms, quality of life, and patient satisfaction. This is particularly relevant in real-world settings, where access to timely sleep studies may be limited due to cost, availability, or logistical barriers. In such cases, well-designed questionnaires offer a practical and patient-centered tool to provide additional valuable information in the diagnostic process and monitor treatment response. Patients frequently face substantial delays due to limited availability of sleep labs, long wait-lists, and insurance hurdles, which can span several months to over a year in some regions [14].

In qualitative studies, patients described sleep testing as inconvenient and costly, further deterring timely evaluation and treatment. These practical barriers underscore the value of validated questionnaires as patient-reported outcome measures, especially in settings where objective testing is not immediately feasible [15].

Excessive daytime sleepiness is among the markers commonly associated with OSA [16]. ESS is the most commonly utilized questionnaire for the individual to subjectively quantify their daytime sleepiness. The questionnaire asks the individual to rate the chance of dozing on a scale of 0 (as none) to 3 (as a high chance) in eight different individual or social circumstances [8]. Based on the scores, the degree of daytime sleepiness and recommendation for seeking medical attention are determined. While our dataset demonstrated a significant improvement in the ESS scores with the surgical treatment for snoring and OSA, there were no correlations between ESS and any of the sleep parameters. This could be due to this questionnaire’s target of daytime sleepiness being more associated with moderate to severe OSA. By contrast, the population of our study represented a spectrum of snorers and subjects with mild to moderate OSA. Statistical significance in the change in ESS score with treatment was evident in higher BMI categories of 25–29.9 and 30+ (Table 9). However, only the AHI sub-group of 5–14.9 was approaching but not reaching significance in higher BMI, probably due to the small sample size (Table 10). On the other hand, in the literature, several other factors have been blamed for the lack of accuracy and high variability between individuals, including lack of education or literacy, ambiguity in the terms, differences in culture and lifestyles of the individuals filling this form [17]. A review comparing eight questionnaires, including PSQI, found that ESS had good internal consistency and construct validity, while the main challenges were in its factorial structure, known-group difference, and estimation of reliable cut-offs [18].

The self-administered scale of VAS is used for subjective feelings of a number of variables in different disciplines, including sleep medicine. A study comparing ESS and VAS before and after positive airway pressure (PAP) treatment demonstrated a better detection of change in sleepiness with VAS [9]. These results were consistent with ours, demonstrating a higher level of significance in demonstrating changes before and after the surgery using VAS. Although the VAS scale is utilized in the field of snoring and OSA for several variables, we only used it to quantify snoring. Obviously, this was limited due to the reliability of the feedback the individual was receiving from others. The huge variability of quality and quantity in the sources of feedback between the individuals weakened the value of VAS as a reliable and comparable sleep parameter. Assuming that the source remains the same, VAS assessment for snoring may have a value when comparing the change with treatment in the same study population. In order to reduce the potential subjectivity and variability of the ESS, Alqurashi et al. compared ESS with self-administered VAS [9]. The questionnaire was constructed to measure sleepiness by instructing the participants to mark on the 100 mm line with the ends marked as “I never fall asleep” and “I always fall asleep”. VAS was used to show, on average, over the past week, how high the likelihood is of the individual falling asleep during the day. It was concluded that VAS was sensitive to change and easier to use compared to ESS [9]. In our study, snoring assessed with VAS demonstrated the highest significance in change with the treatment. Even though there were no correlations between VAS and other sleep parameters before the treatment (Table 5), post-operative VAS for snoring was correlated with the post-op values and the change in LOS and TID < 90% (Table 6 and Table 7).

Health-related quality of life assessment is an important indicator of broad physical and mental health. SF-36 is commonly used for this purpose. Barile et al. analyzed data from 66,269 adult Medicare advantage members age 65 and older, comparing SF-36 with CDC Healthy Days items [10]. Even though it is not specific for snoring or OSA due to known associations and impacts of these conditions on general health, we included the SF-36 assessment in our study. Interestingly, SF-36 was the only questionnaire among the 5 included in our study that did not show a statistically significant change with the treatment. Similarly, SF-36 was not correlated with any of the objective sleep parameters. On the other hand, pre-operatively, SF-36 was correlated with STOP-Bang and PSQI, whereas post-operatively, significant correlations were present with all four other questionnaires.

An easy-to-remember and use questionnaire that combined subjective and objective items was established and validated to screen for OSA in the surgical population. Chung et al. validated the STOP questionnaire in comparison with polysomnography [19]. Our results also demonstrated a significant correlation between the STOP-Bang scores and AHI, both before and after the surgery. Comparison with the Berlin questionnaire (BQ) and Sleep Apnea of Sleep Disorder questionnaire in Korean patients with suspected OSA revealed high sensitivity but lower than acceptable specificity of the STOP-Bang questionnaire. The authors concluded that, despite being useful, better sleep questionnaires need to be developed [20]. A meta-analysis confirmed the successful role of the STOP-Bang questionnaire in screening, identifying, and assessing the severity of OSA [21]. The population of our study included subjects with non-OSA and mild OSA with a pre-operative STOP-Bang score of 3.65+/−1.13 (median 4), which is considered a low score for this tool. In spite of that fact, the change in this score with the surgical treatment was highly significant. This questionnaire demonstrated the highest correlations with the sleep parameters, compared to all five tools that were studied. Pre-operatively, STOP-Bang correlated with AHI and ODI, and post-operatively, with the above as well as with LOS. Compared to the items of BMI, age, neck circumference, and gender (Bang items), the association and change would be expected to be in the STOP-Bang items in the questionnaire: snoring, tiredness, observed apnea, and high blood pressure. STOP-Bang questionnaire is reported to successfully categorize OSA severity and triage patients for further diagnostic evaluation [22]. The sensitivity of the STOP-Bang score ≥ 3 to detect moderate to severe OSA and severe OSA is reported to be 93% and 100%, respectively. For these diagnoses, the negative predictive values were found to be 90% and 100%, respectively [23]. Authors suggested that a STOP-Bang score of 0 to 2 can be classified as low risk, and a score of 5 to 8 can be classified as high risk of moderate to severe OSA. The scores at the midrange (3 or 4) required additional criteria for risk assessment. A systematic review and meta-analysis demonstrated that the STOP-Bang questionnaire is a valid and effective screening tool for OSA in the general population and commercial drivers [24].

Assessment of sleep quality was the goal in developing PSQI [12]. Recognizing that sleep quality was a complex phenomenon that was difficult to define and measure objectively, assessing seven specific components was targeted, including: subjective sleep quality, sleep latency, sleep duration, habitual sleep efficiency, sleep disturbances, use of sleeping medications, and daytime dysfunction. In addition to the 19 self-rated questions, five questions were rated by the bed partner or roommate, if present. In our study, there was a statistically significant improvement in the PSQI scores with the surgical treatment. Subgroup analysis showed the significance in moderate and high BMI, as well as in the two middle AHI categories. However, its correlation with the objective sleep parameters was limited by pre-operative ST.

There are a large number of questionnaires in many different medical disciplines. However, only some have been broadly used outside of the institutions that developed and validated them. Commonly used questionnaires were compared in high-risk populations, and STOP-Bang was identified as superior to OSA50 and BQ despite all limitations [25]. In that study, authors compared five questionnaires (ESS, STOP-Bang, STOP, BQ, and PSQI) in terms of reliability of OSA detection [26]. While STOP-Bang, BQ, and STOP questionnaires had the highest sensitivity, ESS, STOP-Bang, and BQ had the highest specificity. Authors conclude that STOP-Bang and BQ were most suitable for OSA screening, whereas ESS and PSQI were unsuitable. A meta-analysis of clinical screening tests for OSA suggested that most of the clinical screening tests will miss a significant proportion of patients with OSA [27]. A systematic review of screening questionnaires for OSA suggested that STOP and STOP-Bang have higher methodological quality and easy-to-use features [28]. Results of our study also supported a more significant correlation between sleep parameters and STOP-Bang. The usefulness of modifications in the STOP-Bang questionnaire in seniors was explored, compared with AHI, home sleep testing, ESS, and the Athens Insomnia Scale [29]. Authors conclude that the high prevalence of OSA in that population made the use of solely questionnaires inadequate, implying the need for adding objective tests in the first step of screening. A comparison of nine screening questionnaires, including BQ, modified BQ, STOP, STOP-Bang, OSA50, sleep apnea score (SACS), and ESS to assess the likelihood of OSA found SACS and STOP-Bang to have the best positive and negative likelihood ratios [30].

In addition to screening purposes, sleep questionnaires have been used to assess the treatment outcomes. A study compared continuous PAP treatment with mandibular advancement splint device treatment in patients with non-severe OSA. SF-36 was used for health quality, and PSQI to evaluate sleep quality [31]. Authors reported similar improvements in both questionnaires and moderate correlations between SF-36 and PSQI in both treatment methods. We also found that these questionnaires were significantly correlated, while post-operatively, this correlation was more significant (*p*: 0.0002). Moreover, in our study, all questionnaires (ESS, VAS, PSQI, and STOP-Bang) except SF-36 showed statistically significant improvements after the surgical treatment. It suggests that these questionnaires may be useful for assessing treatment outcomes and monitoring long-term changes. Assessing daytime sleepiness by using ESS and polysomnography was the main outcome measured in evaluating the surgical success for OSA in non-obese patients. Significant improvement in ESS scores in mild, moderate, and severe OSA groups was observed [32]. Our study demonstrated a significant improvement in ESS in only mild OSA group. A study on the effectiveness of pharyngoplasty with dorsal palatal flap expansion for OSA demonstrated a statistically significant improvement in VAS and ESS, but not SF-36 [33]. The current study demonstrated similar results. Another study investigating the long-term outcomes of uvulopalatopharyngoplasty or expansion sphincteropharyngoplasty showed an ongoing statistically significant improvement of snoring VAS but not ESS after an average of 96.8+/−30.2 months [34]. Compared to that study on more invasive surgery outcomes, our current study was on minimally invasive surgery, and demonstrated significant improvements in both ESS and VAS.

In other studies, questionnaires have been used to assess the outcomes in populations that had indications and procedures similar to our study. One recent randomized, placebo-controlled study has shown a statistically significant reduction in snoring with RF surgery of the soft palate over placebo. Moreover, VAS reduced from 8.1 to 5.2 in the RF group compared with 8.4 to 8.0 in the placebo group [35]. Our study has demonstrated a similar change in VAS with surgery, decreasing from median of 9 to 4. A review of treatment efficacy was performed utilizing questionnaires including VAS and ESS for the treatment of snoring with office-based procedures such as laser-assisted uvulopalatoplasty, RF, injection snoreplasty, and palatal implants, favored RF over other modalities [36]. Our study did not compare the different treatment modalities; however, it showed significant sensitivity of both VAS and ESS in detecting changes with the surgical treatment. Effect of minimally invasive surgery on sleep quality utilizing ESS and workability, in another study, demonstrated a significant benefit in a study conducted by Zhang [37]. Our study was also on minimally invasive surgical outcomes, and similarly demonstrated significant changes in AHI, ESS and LOS. On the other hand, a positive (continuous PAP) and negative (sham RF surgery) controlled study on patients with mild to moderate OSA receiving RF tongue and palate reduction demonstrated a discordance between the subjective measures, including SF-36, ESS, and the objective measures from polysomnography [38]. Similarly, our study demonstrated a poor correlation between the objective sleep parameters and subjective sleep parameters, before and after the surgery, particularly with ESS.

Based on the results of our study, most questionnaires, except STOP-Bang, were not able to detect the presence and severity of snoring compared to the objective sleep study parameters; therefore, this does not support their role as a screening tool. Even though all questionnaires, except SF-36, had a significant change with treatment, as an outcome assessment tool, only STOP-Bang and snoring VAS were correlated with the sleep study parameters. Snoring VAS was the only questionnaire that was correlated with the magnitude of change in two objective parameters: LOS and TID < 90%. We attribute some of the inconclusive results to the relatively small sample size, an unbalanced distribution of categories of gender, BMI, and AHI in this study sample. Moreover, each questionnaire has specific intent, content, strengths, and weaknesses. Their level of sensitivity and specificity may be dependent on the study population and intervention. Expansion of a database, dissection of each questionnaire, and analysis of specific sections and questions may provide a better understanding. Furthermore, it may answer ongoing questions and may facilitate the construction of better questionnaires for snoring and OSA, either universal or specific to a population, presentation, or the problem, its severity, and/or intended intervention.

Weaknesses of our study included relatively small sample size, absence of a control group, having utilized the Type III sleep study instead of polysomnography, an inclusion of the population of patients suitable for outpatient minimally invasive surgical treatment, an inclusion of patients with non-OSA that needed surgery for snoring, and low number of patients with moderate and severe OSA. The study limitation also applies to the gender imbalance in the cohort, with a predominance of male participants (39 males and 10 females). This also reflects the higher prevalence of OSA in males, but may reduce the generalizability of the findings to female patients. A small number of female participants limits our ability to draw robust conclusions about potential sex-specific differences, which are known to exist in OSA presentation and outcomes. However, our current study was not focused on these differences. Future studies with more balanced gender representation will be important to validate and extend our findings. Therefore, expansion of this dataset will facilitate a better assessment of the sensitivity and specificity of the sleep questionnaires, their value in screening, and the diagnostic and prognostic role in the population of patients with snoring and OSA.

Future research can extend our work in several key directions. First, expanding the dataset to include a more diverse population will improve the generalizability of the findings. Second, incorporating long-term follow-up would also be valuable in evaluating the durability of surgical outcomes and the predictive utility of sleep questionnaires over time. Third, integrating additional sleep parameters obtained from more sleep study reports may help validate and refine subjective questionnaire tools. Moreover, exploring the differential impact of minimally invasive surgery across varying severities of OSA and demographic groups (e.g., age, sex, BMI, comorbidities) might bring new data related to treatment responsiveness. The development of a diagnostic model combining subjective and objective parameters may lead to more personalized and efficient clinical pathways for managing snoring and OSA.

## 5. Conclusions

Sleep questionnaires are a useful part of the workup of snoring and sleep apnea for screening, diagnosis, and monitoring the outcomes of a treatment. When compared to objective sleep parameters, there are differences between questionnaires regarding their ability to identify the presence of snoring and OSA, quantify the severity of snoring and OSA, and predict the changes with treatment for snoring and OSA. Future studies are needed to improve the sensitivity and specificity of questionnaires for snoring and OSA.

## Figures and Tables

**Table 1:** Pre-operative sleep parameters.

Pre-Operative Sleep Parameters	Number of Subjects (*n*)	Average	Standard Deviation	Minimum	Q1	Median	Q3	Maximum
ESS	49	9.04	5.76	0	4.5	9	13	22
VAS	49	8.18	1.93	3	7	9	10	10
SF36	49	42.12	22.86	13	27.5	35	55.5	124
STOP-Bang	49	3.65	1.13	1	3	4	4	6
PSQI	49	6.61	3.23	1	4	6	9	15
AI	49	3.96	4.34	0	0.85	2.2	5.9	19
HI	49	7.86	5.52	0.6	3.25	7.2	11.5	22.7
AHI	49	16.71	9.31	2.1	9	14.7	23.25	40.8
MOS	49	94.33%	1.30%	90%	94%	94%	95%	97%
LOS	49	83.47%	7.36%	55%	80%	86%	88%	93%
ODI	49	14.43	9.13	1.2	7.3	12.4	21.85	37.1
ST	49	17.26%	14.41%	0.5%	4.6%	13.9%	28.2%	49.1%
TID	49	2.30%	5.65%	0.0%	0.2%	0.6%	2.7%	37.6%

Abbreviations = ESS: Epworth sleepiness scale; VAS: visual analog scale for snoring loudness; SF-36: short-form health survey 36; STOP-Bang: “snoring, tiredness, observed apnea, high blood pressure, body mass index, age, neck circumference, and male gender” questionnaire; PSQI: Pittsburgh quality of sleep index; AI: apnea index; HI: hypopnea index; AHI: apnea and hypopnea index; MOS: mean oxygen saturation; LOS: Lowest oxygen saturation; ODI: oxygen desaturation index; ST: snoring time; TID: time in the saturation below 90%.

**Table 2:** Post-operative sleep parameters.

Post-Operative Sleep Parameters	Number of Subjects (*n*)	Average	Standard Deviation	Minimum	Q1	Median	Q3	Maximum
ESS	49	6.61	4.55	0	3	6	10	21
VAS	49	4.13	2.87	0	2	4	6.5	10
SF36	49	42.45	24.70	13	26	34	54	113
STOP-Bang	49	2.49	1.42	0	1	2	3	6
PSQI	49	4.98	2.13	1	3	5	6	10
AI	49	2.31	3.09	0	0.4	1.1	2.9	15.5
HI	49	4.51	3.66	0	1.4	3.3	6.95	16.5
AHI	49	10.39	7.86	1.1	3.6	8.3	16.2	28.5
MOS	49	94.69%	1.50%	91%	94%	95%	96%	98%
LOS	49	85.20%	5.45%	70%	82%	86%	90%	94%
ODI	49	10.17	7.78	0.5	4.15	8.3	14.35	32.7
ST	49	12.55%	13.36%	0.0%	2.9%	8.7%	18.2%	67.8%
TID	49	1.88%	3.23%	0.0%	0.0%	0.5%	1.7%	13.1%

Abbreviations = ESS: Epworth sleepiness scale; VAS: visual analog scale for snoring loudness; SF-36: short-form health survey 36; STOP-Bang: “snoring, tiredness, observed apnea, high blood pressure, body mass index, age, neck circumference, and male gender” questionnaire; PSQI: Pittsburgh quality of sleep index; AI: apnea index; HI: hypopnea index; AHI: apnea and hypopnea index; MOS: mean oxygen saturation; LOS: Lowest oxygen saturation; ODI: oxygen desaturation index; ST: snoring time; TID: time in the saturation below 90%.

**Table 3:** Change in the subjective sleep parameters with surgery.

Subjective Sleep Parameters	Pre-Operative			Post-Operative			Statistical Significance (*p* *)
	Q1	Median	Q3	Q1	Median	Q3	
ESS	4.5	9	13	3	6	10	**0.0003**
VAS	7	9	10	2	4	6.5	**0.0000001**
SF-36	27.5	35	55.5	26	34	54	0.809
PSQI	3	4	4	1	2	3	**0.0004**
STOP-Bang	4	6	9	3	5	6	**0.000004**

* Wilcox pairwise order test: *p* < 0.01 (Bonferroni correction applied). Abbreviations = ESS: Epworth sleepiness scale; VAS: visual analoganalog scale for snoring loudness; SF-36: short-form health survey 36; STOP-Bang: “snoring, tiredness, observed apnea, high blood pressure, body mass index, age, neck circumference, and male gender” questionnaire; PSQI: Pittsburgh quality of sleep index; Q1: range of first quartile from median; Q3: range of third quartile from the median.

**Table 4:** Change in the objective sleep parameters with surgery.

Objective Sleep Parameters	Pre-Operative			Post-Operative			Statistical Significance (*p* *)
	Q1	Median	Q3	Q1	Median	Q3	
AI	0.85	2.2	5.9	0.4	1.1	2.9	0.018
HI	3.25	7.2	11.5	1.4	3.3	6.95	**0.00005**
AHI	9	14.7	23.25	3.6	8.3	16.2	**0.000002**
MOS	94%	94%	95%	94%	95%	96%	0.113
LOS	80%	86%	88%	82%	86%	90%	0.400
ODI	7.3	12.4	21.85	4.15	8.3	14.35	**0.0004**
ST	5%	14%	28%	2.9%	8.7%	18.2%	0.085
TID	0%	1%	3%	0.0%	0.5%	1.7%	0.394

* Wilcox pairwise order test: *p* < 0.00625 (Bonferroni correction applied). Abbreviations = ESS: Epworth sleepiness scale; VAS: visual analoganalog scale for snoring loudness; SF-36: short-form health survey 36; STOP-Bang: “snoring, tiredness, observed apnea, high blood pressure, body mass index, age, neck circumference, and male gender” questionnaire; PSQI: Pittsburgh quality of sleep index; Q1: range of first quartile from median; Q3: range of third quartile from the median.

**Table 5:** Correlations between the subjective and objective sleep parameters before the surgery.

Correlations Before The Surgery		AHI	MOS	LOS	ODI	ST	TID
**ESS**	**r**	0.07	0.00	−0.04	0.12	0.07	0.17
	***p***	0.624	0.985	0.801	0.414	0.624	0.235
**VAS**	**r**	−0.02	0.10	0.04	−0.02	0.17	−0.04
	***p***	0.902	0.478	0.786	0.898	0.257	0.763
**SF36**	**r**	−0.01	0.04	0.20	−0.06	−0.38	−0.07
	***p***	0.932	0.764	0.168	0.694	**0.007**	0.630
**STOP-Bang**	**r**	0.29	−0.23	−0.01	0.34	−0.21	0.14
	***p***	**0.045**	0.106	0.951	**0.017**	0.139	0.338
**PSQI**	**r**	0.08	−0.09	−0.15	0.06	−0.35	0.11
	***p***	0.607	0.531	0.312	0.661	**0.014**	0.469

Statistical significance (non-parametric Spearman correlation analysis): *p* < 0.05. Abbreviations = *r* represents the strength and direction of the linear relationship between the two variables, *p*—probability of observed effect occurring by chance, ESS: Epworth sleepiness scale; VAS: visual analog scale for snoring loudness; SF-36: short-form health survey 36; STOP-Bang: “snoring, tiredness, observed apnea, high blood pressure, body mass index, age, neck circumference, and male gender” questionnaire; PSQI: Pittsburgh quality of sleep index; Q1: range of first quartile from median; Q3: range of third quartile from the median.

**Table 6:** Correlations between the subjective and objective sleep parameters after the surgery.

Correlations After the Surgery		AHI	MOS	LOS	ODI	ST	TID
**ESS**	**r**	0.03	0.05	−0.16	−0.01	0.18	0.09
	***p***	0.853	0.718	0.262	0.938	0.220	0.554
**VAS**	**r**	0.26	−0.14	−0.32	0.27	0.08	0.29
	***p***	0.069	0.348	**0.025**	0.058	0.568	**0.043**
**SF36**	**r**	−0.05	−0.11	−0.05	−0.10	−0.14	0.02
	***p***	0.715	0.463	0.724	0.509	0.328	0.900
**STOP-Bang**	**r**	0.30	−0.26	−0.35	0.31	0.09	0.26
	***p***	**0.034**	0.072	**0.015**	**0.030**	0.521	0.068
**PSQI**	**r**	−0.06	−0.03	−0.02	−0.08	−0.26	−0.10
	***p***	0.684	0.812	0.911	0.575	0.072	0.484

Statistical Significance (non-parametric Spearman correlation analysis): *p* < 0.05. Abbreviations: *r* represents the strength and direction of the linear relationship between the two variables, *p*—probability of observed effect occurring by chance, ESS: Epworth sleepiness scale; VAS: visual analog scale for snoring loudness; SF-36: short-form health survey 36; STOP-Bang: “snoring, tiredness, observed apnea, high blood pressure, body mass index, age, neck circumference, and male gender” questionnaire; PSQI: Pittsburgh quality of sleep index; AI: apnea index; HI: hypopnea index; AHI: apnea and hypopnea index; MOS: mean oxygen saturation; LOS: Lowest oxygen saturation; ODI: oxygen desaturation index; ST: snoring time; TID: time in the saturation below 90%.

**Table 7:** Correlations between the pre-operative subjective sleep parameters and the magnitude of change in the objective sleep parameters with the surgical treatment.

Correlations Between Pre-Op and The Change with Surgery		AHI	MOS	LOS	ODI	ST	TID
**ESS**	**r**	−0.07	−0.01	0.06	−0.08	0.09	−0.01
	***p***	0.620	0.939	0.700	0.562	0.536	0.952
**VAS**	**r**	0.15	−0.26	−0.31	0.20	0.00	0.30
	***p***	0.318	0.077	**0.033**	0.170	0.993	**0.037**
**SF36**	**r**	−0.09	−0.09	0.00	0.06	0.26	0.25
	***p***	0.533	0.551	0.991	0.673	0.073	0.080
**STOP_Bang**	**r**	−0.05	−0.06	−0.18	0.01	0.13	0.19
	***p***	0.742	0.674	0.207	0.952	0.385	0.196
**PSQI**	**r**	−0.07	0.11	0.18	−0.06	0.27	−0.14
	*p*	0.614	0.471	0.218	0.659	0.065	0.345

Statistical significance (non-parametric Spearman correlation analysis): *p* < 0.05. Abbreviations: *r* represents the strength and direction of a linear relationship between the two variables, *p*—probability of observed effect occurring by chance, ESS: Epworth sleepiness scale; VAS: visual analog scale for snoring loudness; SF-36: short-form health survey 36; STOP-Bang: “snoring, tiredness, observed apnea, high blood pressure, body mass index, age, neck circumference, and male gender” questionnaire; PSQI: Pittsburgh quality of sleep index; AI: apnea index; HI: hypopnea index; AHI: apnea and hypopnea index; MOS: mean oxygen saturation; LOS: Lowest oxygen saturation; ODI: oxygen desaturation index; ST: snoring time; TID: time in the saturation below 90%.

**Table 8:** Gender specific differences in the sleep parameters before and after the surgery and the changes with the surgery.

	Gender	Subject Number (*n*)	Pre-Operative		Post-Operative		*p* *
			Median	*p* **	Median	*p* **	
ESS	Female	10	10	0.494	5.5	0.756	**0.021**
	Male	39	9		6		**0.005**
VAS	Female	10	6	**0.004**	3	0.388	**0.014**
	Male	39	9		4		**0.000**
SF36	Female	10	37.5	0.611	38.5	0.188	0.139
	Male	39	34		32		0.264
STOP-Bang	Female	10	3	0.052	2	0.395	0.176
	Male	39	4		2		**0.000**
PSQI	Female	10	8	0.336	4.5	0.909	0.073
	Male	39	6		5		**0.002**
AHI	Female	10	9	**0.015**	4.3	0.058	0.059
	Male	39	17.1		9		**0.000**
MOS	Female	10	95.00%	0.407	94.50%	0.859	0.516
	Male	39	94.00%		95.00%		0.152
LOS	Female	10	84.00%	0.325	86.50%	0.279	0.123
	Male	39	86.00%		85.00%		0.966
ODI	Female	10	6.6	**0.010**	5	**0.021**	0.233
	Male	39	14		9.7		**0.001**
ST	Female	10	14.10%	0.843	6.15%	0.365	0.074
	Male	39	13.90%		9.30%		0.365
TID	Female	10	0.65%	0.804	0.05%	0.066	0.052
	Male	39	0.50%		0.50%		0.941

* Wilcoxon Signed Ranks Test, ** Mann–Whitney/Kruskal-allis test: *p* < 0.05. Abbreviations = ESS: Epworth sleepiness scale; VAS: visual analog scale for snoring loudness; SF-36: short-form health survey 36; STOP-Bang: “snoring, tiredness, observed apnea, high blood pressure, body mass index, age, neck circumference, and male gender” questionnaire; PSQI: Pittsburgh quality of sleep index; AI: apnea index; HI: hypopnea index; AHI: apnea and hypopnea index; MOS: mean oxygen saturation; LOS: lowest oxygen saturation; ODI: oxygen desaturation index; ST: snoring time; TID: time in the saturation below 90%.

**Table 9:** Body mass index (BMI) category-specific differences in the sleep parameters before and after the surgery and the changes with the surgery.

	BMI Category	Subject Number (*n*)	Pre-Operative		Post-Operative		*p* *
			Median	*p* **	Median	*p* **	
ESS	<25.0	14	9	0.108	4.5	0.850	0.099
	25.0–29.9	26	7.5		6		**0.034**
	30.0+	9	12		7		**0.028**
VAS	<25.0	14	8.5	0.919	3.5	0.687	**0.002**
	25.0–29.9	26	9		4		**0.0003**
	30.0+	9	8		3		**0.008**
SF36	<25.0	14	37.5	0.192	35.5	0.600	0.701
	25.0–29.9	26	33		31.5		0.949
	30.0+	9	54		41		0.407
STOP-Bang	<25.0	14	3.5	0.088	2	0.869	0.056
	25.0–29.9	26	4		2		**0.0005**
	30.0+	9	4		3		**0.010**
PSQI	<25.0	14	5	0.277	5.5	0.202	0.502
	25.0–29.9	26	7		5		**0.007**
	30.0+	9	6		4		**0.007**
AHI	<25.0	14	10.95	0.321	6.2	0.481	**0.008**
	25.0–29.9	26	14.65		7.85		**0.0003**
	30.0+	9	17.8		10.2		0.066
MOS	<25.0	14	95.00%	**0.042**	95.00%	0.408	0.791
	25.0–29.9	26	94.00%		94.50%		0.127
	30.0+	9	94.00%		94.00%		0.495
LOS	<25.0	14	88.00%	0.076	87.50%	0.360	0.227
	25.0–29.9	26	86.00%		85.50%		0.855
	30.0+	9	80.00%		83.00%		0.720
ODI	<25.0	14	7.85	0.091	4.75	0.120	0.050
	25.0–29.9	26	12.7		8.25		**0.004**
	30.0+	9	15.6		13.7		0.236
ST	<25.0	14	7.60%	0.844	8.50%	0.266	0.221
	25.0–29.9	26	12.95%		7.20%		0.158
	30.0+	9	15.60%		16.50%		0.859
TID	< 25.0	14	0.50%	0.177	0.30%	0.494	0.087
	25.0–29.9	26	0.40%		0.40%		0.943
	30.0+	9	2.50%		1.40%		0.833

* Wilcoxon Signed Ranks Test, ** Mann–Whitney/Kruskal-allis test: *p* < 0.05. Abbreviations = ESS: Epworth sleepiness scale; VAS: visual analog scale for snoring loudness; SF-36: short-form health survey 36; STOP-Bang: “snoring, tiredness, observed apnea, high blood pressure, body mass index, age, neck circumference, and male gender” questionnaire; PSQI: Pittsburgh quality of sleep index; AI: apnea index; HI: hypopnea index; AHI: apnea and hypopnea index; MOS: mean oxygen saturation; LOS: Lowest oxygen saturation; ODI: oxygen desaturation index; ST: snoring time; TID: time in the saturation below 90%.

**Table 10:** Apnea hypopnea index (AHI) category-specific differences in the sleep parameters before and after the surgery, and the changes with the surgery.

	AHI Category	Subject Number (*n*)	Pre-Operative		Post-Operative		*p* *
			Median	*p* **	Median	*p* **	
ESS	<5.0	5	10	0.814	7	0.801	0.416
	5.0–14.9	20	7.5		6		**0.009**
	15.0–29.9	20	9.5		4.5		0.069
	30.0+	4	12.5		7.5		0.066
VAS	<5.0	5	7	0.221	2	0.423	0.080
	5.0–14.9	20	10		4		**0.001**
	15.0–29.9	20	8		3.5		**0.000**
	30.0+	4	8		4		0.068
SF36	<5.0	5	39	0.810	51	0.679	**0.043**
	5.0–14.9	20	35		30		0.825
	15.0–29.9	20	35.5		33.5		0.218
	30.0+	4	33		33.5		0.715
STOP-Bang	<5.0	5	3	0.155	2	0.257	0.059
	5.0–14.9	20	4		3		**0.033**
	15.0–29.9	20	4		2		**0.001**
	30.0+	4	3.5		3		0.102
PSQI	<5.0	5	4	0.363	4	0.923	0.593
	5.0–14.9	20	7.5		5		**0.017**
	15.0–29.9	20	6		5		**0.033**
	30.0+	4	7.5		5		0.066
AHI	<5.0	5	4	**0.00000001**	2.4	**0.0003**	0.500
	5.0–14.9	20	10.65		5		**0.021**
	15.0–29.9	20	22.55		12.8		**0.000**
	30.0+	4	36.25		17.65		0.068
MOS	< 5.0	5	96.00%	**0.042**	96.00%	0.062	0.414
	5.0–14.9	20	94.05%		94.00%		0.391
	15.0–29.9	20	94.00%		94.00%		0.541
	30.0+	4	94.00%		96.00%		0.276
LOS	< 5.0	5	89.00%	**0.010**	92.00%	**0.015**	0.157
	5.0–14.9	20	83.00%		84.00%		0.420
	15.0–29.9	20	86.00%		86.00%		0.981
	30.0+	4	80.00%		81.00%		1.000
ODI	<5.0	5	2.9	**0.00000001**	1.2	**0.001**	0.279
	5.0–14.9	20	9.4		5.85		0.191
	15.0–29.9	20	20.2		13.75		**0.001**
	30.0+	4	32		14.5		0.144
ST	<5.0	5	30.50%	0.552	3.30%	0.437	**0.043**
	5.0–14.9	20	13.75%		9.10%		0.263
	15.0–29.9	20	10.45%		9.95%		0.936
	30.0+	4	17.25%		9.75%		0.465
TID	<5.0	5	0.20%	**0.036**	0.00%	0.129	0.500
	5.0–14.9	20	0.40%		0.15%		0.485
	15.0–29.9	20	0.60%		0.70%		0.862
	30.0+	4	3.90%		2.35%		0.273

* Wilcoxon Signed Ranks Test, ** Mann–Whitney/Kruskal-allis test: *p* < 0.05. Abbreviations = ESS: Epworth sleepiness scale; VAS: visual analog scale for snoring loudness; SF-36: short-form health survey 36; STOP-Bang: “snoring, tiredness, observed apnea, high blood pressure, body mass index, age, neck circumference, and male gender” questionnaire; PSQI: Pittsburgh quality of sleep index; AI: apnea index; HI: hypopnea index; AHI: apnea and hypopnea index; MOS: mean oxygen saturation; LOS: lowest oxygen saturation; ODI: oxygen desaturation index; ST: snoring time; TID: time in the saturation below 90%.

**Table 11:** Correlations between the sleep questionnaires before the surgery.

			Pre-Operative				
			ESS	VAS	SF-36	STOP-Bang	PSQI
PRE-OPERATIVE	ESS	r	1.00	0.10	0.21	0.26	0.15
		*p*		0.477	0.139	0.071	0.293
	VAS	r	0.10	1.00	−0.12	0.10	−0.02
		*p*	0.477		0.427	0.486	0.871
	SF36	r	0.21	−0.12	1.00	0.41	0.42
		*p*	0.139	0.427		**0.004**	**0.003**
	STOP-Bang	r	0.26	0.10	0.41	1.00	0.25
		*p*	0.071	0.486	**0.004**		0.084
	PSQI	r	0.15	−0.02	0.42	0.25	1.00
		*p*	0.293	0.871	**0.003**	0.084	

Statistical Significance (non-parametric Spearman correlation analysis): *p* < 0.05. Abbreviations= *r* represents the strength and direction of the linear relationship between the two variables, *p*—probability of observed effect occurring by chance, ESS: Epworth sleepiness scale; VAS: visual analog scale for snoring loudness; SF-36: short-form health survey 36; STOP-Bang: “snoring, tiredness, observed apnea, high blood pressure, body mass index, age, neck circumference, and male gender” questionnaire; PSQI: Pittsburgh quality of sleep index; AI: apnea index; HI: hypopnea index; AHI: apnea and hypopnea index; MOS: mean oxygen saturation; LOS: Lowest oxygen saturation; ODI: oxygen desaturation index; ST: snoring time; TID: time in the saturation below 90%.

**Table 12:** Correlations between the sleep questionnaires after the surgery.

			Post-Operative				
			ESS	VAS	SF-36	STOP-Bang	PSQI
POST-OPERATIVE	ESS	r	1.00	0.34	0.40	0.41	0.20
		*p*		**0.016**	**0.005**	**0.003**	0.175
	VAS	r	0.34	1.00	0.28	0.69	0.29
		*p*	**0.016**		**0.050**	**0.00000004**	**0.040**
	SF36	r	0.40	0.28	1.00	0.37	0.51
		*p*	**0.005**	**0.050**		**0.010**	**0.0002**
	STOP-Bang	r	0.41	0.69	0.37	1.00	0.36
		*p*	**0.003**	**0.00000004**	**0.010**		**0.011**
	PSQI	r	0.20	0.29	0.51	0.36	1.00
		*p*	0.175	**0.040**	**0.0002**	**0.011**	

Statistical Significance (Non-parametric Spearman correlation analysis): *p* < 0.05. Abbreviations = *r* represents the strength and direction of the linear relationship between the two variables, *p*—probability of observed effect occurring by chance, ESS: Epworth sleepiness scale; VAS: visual analog scale for snoring loudness; SF-36: short-form health survey 36; STOP-Bang: “snoring, tiredness, observed apnea, high blood pressure, body mass index, age, neck circumference, and male gender” questionnaire; PSQI: Pittsburgh quality of sleep index; AI: apnea index; HI: hypopnea index; AHI: apnea and hypopnea index; MOS: mean oxygen saturation; LOS: Lowest oxygen saturation; ODI: oxygen desaturation index; ST: snoring time; TID: time in the saturation below 90%.

## Data Availability

Deidentified data is available for sharing.

## Institutional Board Statement

The study was approved by the local Bioethics Committee (approval no. APK.002.376.2021) and conducted according to GCP (Guidelines for Good Clinical Practice), and approval date on 23 September 2021.

## Abbreviations

OSA	Obstructive sleep apnea
ESS	Epworth sleepiness scale
VAS	Visual analog scale for snoring loudness
SF-36	Short Form Health Survey 36
STOP-Bang	Snoring, tiredness, observed apnea, high blood pressure, body mass index, age, neck circumference, and gender
PSQI	Pittsburgh quality of sleep index
AI	Apnea index
HI	Hypopnea index
AHI	Apnea–hypopnea index
ODI	Oxygen desaturation index
ST	Mean percentage of snoring time
MDT	Multidisciplinary team
GCP	Guidelines for good clinical practice
BMI	Body mass index
MOS	Mean oxygen saturation
LOS	Lowest oxygen saturation
TID	Percent of sleep time with the oxygen saturation below 90%
RF	Radiofrequency
MHz	Megahertz
r	Strength and direction of the linear relationship between the two variables
*p*	Probability of observed effect occurring by chance
BQ	Berlin questionnaire
